# Serum IgA, IgM, and IgG responses in COVID-19

**DOI:** 10.1038/s41423-020-0474-z

**Published:** 2020-05-28

**Authors:** Huan Ma, Weihong Zeng, Hongliang He, Dan Zhao, Dehua Jiang, Peigen Zhou, Linzhao Cheng, Yajuan Li, Xiaoling Ma, Tengchuan Jin

**Affiliations:** 1 Department of Obstetrics and Gynecology, The First Affiliated Hospital of USTC, Division of Life Sciences and Medicine, University of Science and Technology of China, Hefei, 230001 Anhui China; 2 Hefei National Laboratory for Physical Sciences at Microscale, Laboratory of Structural Immunology, CAS Key Laboratory of Innate Immunity and Chronic Disease, Division of Life Sciences and Medicine, University of Science and Technology of China, Hefei, 230027 Anhui China; 3 Department of Infectious Diseases, The First Affiliated Hospital of USTC, Division of Life Sciences and Medicine, University of Science and Technology of China, Hefei, 230001 Anhui China; 4 Kangrun Biotech LTD, Guangzhou, 511400 Guangdong China; 5 Department of Statistics, University of Wisconsin-Madison, Madison, WI 53706 USA; 6 Division of Life Sciences and Medicine, University of Science and Technology of China, Hefei, 230027 Anhui China; 7 Johns Hopkins University School of Medicine, Columbia, MD USA; 8 Department of Clinical Laboratory, The First Affiliated Hospital of Anhui Medical University, Hefei, 230032 China; 9 Department of Laboratory Medicine, The First Affiliated Hospital of USTC, Division of Life Sciences and Medicine, University of Science and Technology of China, Hefei, 230001 Anhui China

**Keywords:** Infection, Viral infection

Currently, detecting SARS-CoV-2 RNAs is a standard approach for COVID-19 diagnosis. However, there is an urgent need for reliable and rapid serological diagnostic methods to screen SARS-CoV-2-infected people including those who do not have overt symptoms. Most emerging studies described serological tests based on detection of SARS-CoV-2-specific IgM and IgG.^1–4^ Although detection of SARS-CoV-2-specific IgA in serum has been reported in few papers,^5,6^ analyses of IgA levels in a larger number of COVID-19 patients are still lacking.

This study enrolled a total of 87 confirmed COVID-19 patients (Supplementary Table 1) who were admitted to the First Affiliated Hospital of USTC Hospital or the First Affiliated Hospital of Anhui Medical University between January 26, and Mar 5, 2020. Their blood samples were collected during routine clinical testing. All enrolled cases were confirmed with SARS-CoV-2 infection by use of a standard RT-qPCR assay on throat swab samples from the respiratory tract. For all of the enrolled patients, the date of illness onset, clinical classifications of severity, RNA testing results during the hospitalization period, and the personal demographic information were obtained from the clinical records.

Highly purified receptor-binding domain (RBD) of the SARS-CoV-2 spike protein (Supplementary Fig. 1) was expressed in human 293F cells and used to make a set of chemical luminescence kits for detecting the presence of RBD-specific IgA, IgM, and IgG, respectively. To evaluate the diagnostic power of the these kits, 216 sera from 87 SARS-CoV-2-infected patients and a total of 483 control sera including 330 healthy sera, 138 “interfering” sera of other-type patients and 15 sera from once-suspected pneumonia cases were tested. The detected signals relative light units (RLU), for each of isotype of the RBD-specific antibodies, were plotted (Fig. 1a–c). The RBD-specific IgA, IgM, and IgG kits showed diagnostic sensitivities of 98.6%, 96.8%, and 96.8%, and specificities of 98.1%, 92.3%, and 99.8%, respectively (Supplementary Fig. 2a–c). The sensitivities, specificities, and overall agreements of the RBD-specific IgA, IgM, or IgG kit and their combinations are also summarized in Supplementary Table 2. When combining the RBD IgA and IgG kits, the sensitivity, specificity, and overall agreement elevated to 99.1%, 100%, and 99.7%, respectively. This is better than those when IgM and IgG kits are combined using our data or the previous data shown by others.^1–4^

**Fig. 1:**
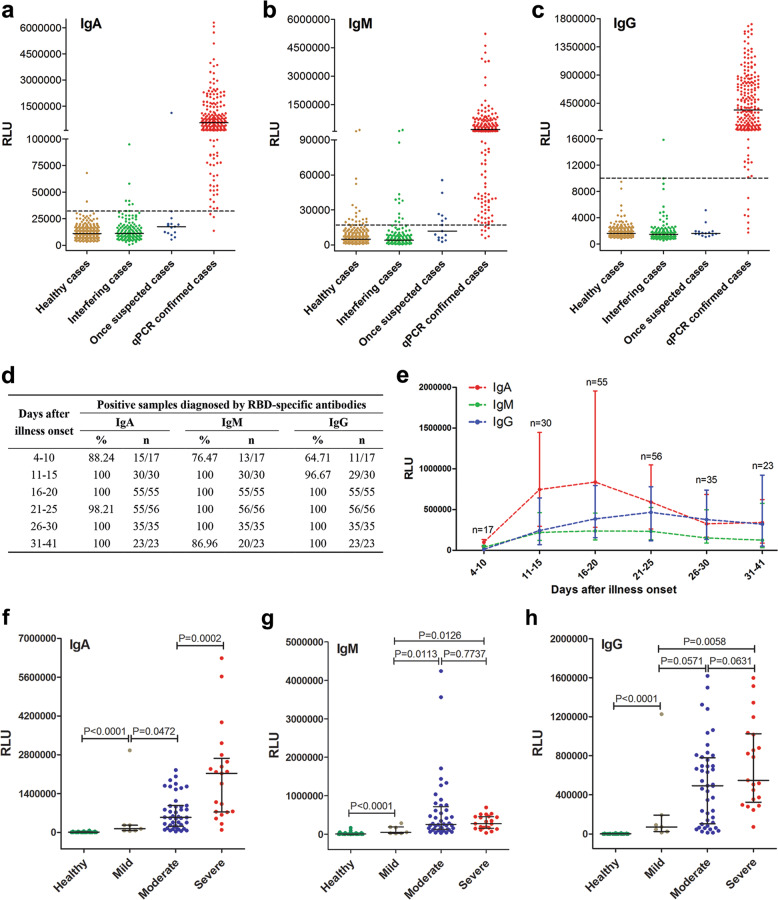
Analysis of SARS-CoV-2 RBD-specific IgA, IgM, and IgG antibodies in 87 COVID-19 patients. Testing results of RBD-specific IgA (**a**), IgM (**b**), and IgG (**c**) kits using 330 healthy sera, 138 sera from other-type of patients who may interfere with the test, 15 sera of once-suspected pneumonia patients, and 216 sera of 87 qPCR-confirmed COVID-19 patients. RLU relative light units. Black bar indicates median values. The dotted line indicates the cut-off value for detecting of each isotypes of antibodies. **d** Sensitivity of RBD-specific IgA, IgM, and IgG detection in serum samples obtained at different periods after illness onset. The kinetics of anti-RBD IgA, IgM, and IgG levels in sera of COVID-19 patients at different time windows was analyzed (**e**). The median values of RLU were plotted for each isotype of three antibodies. Bars indicate median with interquartile ranges. **f**–**h** Serum antibody levels in healthy and three distinct severity groups of COVID-19 patients were analyzed. Healthy: 330 sera; Mild: 7 sera; Moderate: 44 sera; and Severe: 21 sera. The critically ill patients were included into the severe group. Only the data of serum antibody levels at 16–25 days after illness onset of COVID-19 patients were used

In order to investigate the seroconversion during COVID-19 pathogenesis, all the data from 216 sera samples were divided into six groups according to the time windows of collection after illness onset (Fig. 1d). At 4–10 days after symptom onset, the IgA kit exhibited the highest positive diagnostic rate as 88.2% (15/17), while IgM and IgG kit showed detection rates of 76.4% (13/17) and 64.7% (11/17), respectively. The 2 sera diagnosed as negative in the 4–10 days group by IgA kit were collected at the 4th day after illness onset, all other sera includes 2 at the 6th day, 3 at the 7th day, 1 at the 8th day, 6 at the 9th day, and 3 at 10th day after illness onset were tested as positive. In the group of 11–41 days after symptom onset, both RBD IgA and IgG kit showed the same positive diagnostic rate of 99.5% (198/199). In contrast, IgM kit somehow showed a relatively lower positive diagnostic rate as 98.5% (196/199). These results suggest that including IgA in a test provides better diagnostic outcome in early stages. Overall, the medium seroconversion time for IgA, IgM, and IgG are 4–6, 4–6, and 5–10 days post symptom onset, respectively, if tested with the RBD-kits described in this study. While it generally follows a typical seroconversion and immunoglobulin class switching time course, our kits provides an early diagnosis solution due to high sensitivities.

To better understand the trends of antibody levels in all of the 87 COVID-19 patients (some of them contributed multiple samples), we plotted the median RLU reading according the time windows when sera were collected (Fig. 1e). IgA detection shows the highest sensitivity during about 4–25 days after illness onset. The median RLU of RBD-specific IgA reached the peak during 16–20 days after illness onset, and then began to decline but remained at relatively high reading until 31–41 days. The median RLU of RBD-specific IgG was the lowest in early disease stages but raised at 15 days post illness onset, the IgG reached its peak during 21–25 days after illness onset, and stayed at a relatively high reading until 31–41 days, suggesting that IgG is powerful for diagnostics at later stages. Although IgM reached its peak at early stages, the RLU reading was lower than that of IgA or IgG.

We further divided the 87 patients into three severity groups based on established clinical classifications. Consistent with a previous report,^7^ we found that COVID-19 severity is correlated positively with age in our cohort (Supplementary Fig. 3). Patients with severe symptoms were significantly older (median age of 62.5) than those patients with moderate (median age of 46) and mild symptoms (median age of 30), as expected. We used the data of antibody levels at the period of 16–25 days after illness onset, when all of the three isotypes reached or were near their peaks (Fig. 1e). If there were more than one data points, the average value was taken. Serum IgM and IgG levels in moderate and severe COVID-19 patients were significantly higher than mild cases, while no significant difference was observed between severe and moderate patients (Fig. 1g, h). However, we found that IgA levels in severe cases were significantly higher than those mild or moderate cases (Fig. 1f). The molecular mechanism of this observation warrants future studies.

There are some limitations in this study at the current form. We used 216 serum samples from 87 confirmed COVID-19 patients in this study, and serum samples were not available every day for each patient. The earliest serum was collected at the 4th day, and last one was at the 41th day after self-reported illness onset. There are only 17 cases of serum samples collected within the first 10 days after illness onset; which consequently influenced the accuracy. Similarly, there were only 23 cases of serum samples taken after 30 days post illness onset, hampering an analysis of long-term antibody levels in recovered patients. We are currently following up some of the 87 convalescent COVID-19 patients who are willing to participate in further study. Nevertheless, this study provide valuable information regarding COVID-19 serological testing and seroconversion responses, especially for IgA antibodies.

## Supplementary information

Supplemental materials
