# Supplementary material for: Serum IgA, IgM, and IgG responses in COVID-19
Source: Cell Mol Immunol. 2020 May 28;17(7):773–5. doi: 10.1038/s41423-020-0474-z (PMC7331804; doi:10.1038/s41423-020-0474-z)
Supplement: Supplementary file 1 — Supplemental materials [file 41423_2020_474_MOESM1_ESM.doc]

**Supplementary Materials**

**Methods**

**Patients and clinical samples**

This study was reviewed and approved by the Medical Ethical Committee of the First Affiliated Hospital of USTC (approval number: 2020-XG(H)-014) and the First Affiliated Hospital of Anhui Medical University (approval number: Quick-PJ 2020-04-16).

Confirmed COVID-19 cases and clinical classifications were defined according to the New Coronavirus Pneumonia Prevention and Control Program (7th edition) published by the National Health Commission of China. Among the 87 patients, five patients were critical and admitted to the ICU, one was died of cerebral hemorrhage after stroke. Seventeen patients had severe COVID-19, and all of whom required oxygen supplementation. Fifty-six patients had moderate and nine patients had mild COVID-19. The median age of patients was 48 years (range 21–91), and the average age was 47.4 years. Thirty-seven (42.5%) patients had underline illnesses; the most common one was hypertension in 18 patients (20.7%). A total 216 serum samples were taken from the 87 COVID-19 patients, 56 (64.4%), 38 (43.7%), 22 (25.3%) and 10 (11.5%) patients were taken blood for more than 2, 3, 4 and 5 times, respectively.

Sera as negative controls were collected in order to evaluate the reliability of the diagnosis. This cohort contains 330 archived sera from healthy donors (samples collected before October 2019), 138 interfering sera from no-COVID-19 patients with different underlying diseases, and fifteen sera from once suspected cases (RT-qPCR negative but had typical manifestation of pneumonia). All sera were stored at -20°C before use.

**Chemiluminescence immuno-analysis (CLIA) for SARS-CoV-2 diagnosis**

Briefly, the purified RBD viral antigens were coated onto magnetic particles for catching SARS-CoV-2 specific IgA, IgM and IgG in sera. A second antibody that recognizes IgA, IgM or IgG conjugated with acridinium (which can react with substrates to generate a strong chemiluminescence) was used for detecting the IgA, IgM or IgG caught by antigen, respectively. The detected chemiluminescent signal over background signal was calculated as relative light units (RLU). Such collection contains all contents for CLIA of antigen-specific immunoglobulin is called kit here. Serum samples were collected by centrifugation of whole blood in test tubes at room temperature for 15 min. Prior to testing, a denaturant solution was added to each serum to a final concentration of 1% TNBP, 1% Triton X-100. After adequate mixing by inverting, the samples were incubated at 30°C for 4 hours to completely denature any potential viruses. Such solvent/detergent (1% TNBP + 1% Triton X-100) treatment is recommended by WHO guidelines on virus inactivation and removal procedures intended to assure the viral safety of human blood plasma products (https**://www.who.int/bloodproducts**/publications/WHO_TRS_924_A4.pdf). Virus-inactivated serum samples were then diluted 40 times with dilution buffer and subjected to testing at room temperature. Then RLU was measured using a fully automatic chemical luminescent immunoanalyzer, Kaeser 1000 (Kangrun Biotech, Guangzhou, China).

**Statistical analysis**

Based on the clinical RT-qPCR diagnosis results of SARS-CoV-2 infection, receiver operating characteristic (ROC) analysis was conducted using MedCalc software to determine the optimal cut-off value (criterion) and evaluate the diagnostic value of RBD-specific IgA, IgM and IgG detection.

The specificity and sensitivity of the antibody detection were calculated according to the following formulas:

Specificity (%) = 100 x [True negative / (True Negative + False Positive)];

Sensitivity (%) = 100 x [True Positive / (True Positive + False Negative)];

Overall agreement (%) = (True negative + True Positive) / Total tests.

Continuous variables were compared with the Mann-Whitney U test using PRISM version 6.0. A p value less than 0.05 was judged statistically significant.

**Supplementary Tables**

**Supplementary Table 1**. Information of the 87 RT-qPCR confirmed COVID-19 patients.

| **Patients No.** | **Disease severity** | **Underlying disease*** |
| --- | --- | --- |
| 1 | Critical (ICU) | 1, 2, 3 |
| 2 | Critical (ICU) | 4, 5, 6 |
| 3 | Critical (ICU) | 7, 4, 8, 9 |
| 4 | Critical (ICU) | 10, 11 |
| 5 | Critical (ICU) | 5, 12 |
| 6 | Severe | 4 |
| 7 | Severe | none |
| 8 | Severe | none |
| 9 | Severe | 4 |
| 10 | Severe | none |
| 11 | Severe | none |
| 12 | Severe | 4, 13 |
| 13 | Severe | 1 |
| 14 | Severe | none |
| 15 | Severe | none |
| 16 | Severe | 4, 7, 10, 14, 15 |
| 17 | Severe | 4 |
| 18 | Severe | 4 |
| 19 | Severe | 4, 10, 16 |
| 20 | Severe | none |
| 21 | Severe | none |
| 22 | Severe | 17 |
| 23 | Moderate | none |
| 24 | Moderate | none |
| 25 | Moderate | none |
| 26 | Moderate | none |
| 27 | Moderate | 18 |
| 28 | Moderate | none |
| 29 | Moderate | 19, 5 |
| 30 | Moderate | none |
| 31 | Moderate | 20, 21, 22 |
| 32 | Moderate | 4 , 10, 19 |
| 33 | Moderate | none |
| 34 | Moderate | none |
| 35 | Moderate | 19 |
| 36 | Moderate | 4 |
| 37 | Moderate | 10, 1, 23 |
| 38 | Moderate | none |
| 39 | Moderate | none |
| 40 | Moderate | none |
| 41 | Moderate | 19, 20 |
| 42 | Moderate | 4 |
| 43 | Moderate | 24 |
| 44 | Moderate | 25 |
| 45 | Moderate | none |
| 46 | Moderate | none |
| 47 | Moderate | 3, 4, 10, 26 |
| 48 | Moderate | none |
| 49 | Moderate | none |
| 50 | Moderate | none |
| 51 | Moderate | 27 |
| 52 | Moderate | 4, 28 |
| 53 | Moderate | none |
| 54 | Moderate | none |
| 55 | Moderate | 4, 10, 29 |
| 56 | Moderate | none |
| 57 | Moderate | none |
| 58 | Moderate | 4, 30 |
| 59 | Moderate | none |
| 60 | Moderate | none |
| 61 | Moderate | none |
| 62 | Moderate | none |
| 63 | Moderate | none |
| 64 | Moderate | 31, 32 |
| 65 | Moderate | 10 |
| 66 | Moderate | none |
| 67 | Moderate | none |
| 68 | Moderate | 33 |
| 69 | Moderate | none |
| 70 | Moderate | 1 |
| 71 | Moderate | none |
| 72 | Moderate | none |
| 73 | Moderate | none |
| 74 | Moderate | 1 |
| 75 | Moderate | none |
| 76 | Moderate | none |
| 77 | Moderate | none |
| 78 | Moderate | none |
| 79 | Mild | none |
| 80 | Mild | none |
| 81 | Mild | none |
| 82 | Mild | none |
| 83 | Mild | 4, 34 |
| 84 | Mild | 4 |
| 85 | Mild | none |
| 86 | Mild | none |
| 87 | Mild | none |

*****Underlying illness: 1. Hepatitis B; 2. Postoperative hepatocellular carcinoma; 3. Respiratory failure; 4. Hypertension; 5. Cerebral infarction; 6. Upper gastrointestinal bleeding; 7. Coronary heart disease; 8. Cholecystectomy; 9. Benign prostatic hyperplasia; 10. Diabetes; 11. 1. Hepatitis B; 2. Postoperative hepatocellular carcinoma; 3. Respiratory failure; 4. Hypertension; 5. Cerebral infarction; 6. Upper gastrointestinal bleeding; 7. Coronary heart disease; 8. Cholecystectomy; 9. Benign prostatic hyperplasia; 10. Diabetes; 11. Rheumatoid Arthritis; 12. Hernia surgery; 13. Lower extremity arterial occlusion; 14. Heart valve disease; 15. Atrial fibrillation; 16. Expansion; 17. Car accident caused multiple soft tissue, intracranial hemorrhage, and improved after conservative; 18. Chronic gastritis; 19. Hyperlipidemia 20. Leukopenia; 21. Anemia; 22. Sinus tachycardia; 23.Fatty Liver; 24. Proteinuria; 25. Hyperthyroidism; 26. Hypoproteinemia; 27. Uremia; 28. Left nephrectomy; 29. Breast cancer; 30. Parkinson; 31. Frozen shoulder; 32. Rheumatism; 33. Nasal drip syndrome; 34. Hyperuricemia.

**Supplementary Table 2**. Comparisons of sensitivity, specificity and overall agreements of RBD-specific IgA, IgM, and IgG kit and their combinations for diagnosing SARS-CoV-2.

| **Antibody type** | **Sensitivity** | |  | **Specificity** | |  | **Overall agreement** | |
| --- | --- | --- | --- | --- | --- | --- | --- | --- |
|  | **%** | **n/total** |  | **%** | **n/total** |  | **%** | **n/total** |
| IgA | 98.6 | 213/216 |  | 98.1 | 474/483 |  | 98.3 | 687/699 |
| IgM | 96.8 | 209/216 |  | 92.3 | 446/483 |  | 93.7 | 655/699 |
| IgG | 96.8 | 209/216 |  | 99.8 | 482/483 |  | 98.9 | 691/699 |
| IgA and IgM | 95.8 | 207/216 |  | 90.7 | 438/483 |  | 92.3 | 645/699 |
| IgA and IgG | 96.3 | 208/216 |  | 97.9 | 473/483 |  | 97.4 | 681/699 |
| IgM and IgG | 94.9 | 205/216 |  | 92.1 | 445/483 |  | 93.0 | 650/699 |
| IgA and IgM and IgG | 94.4 | 204/216 |  | 90.5 | 437/483 |  | 91.7 | 641/699 |
| IgA or IgM | 99.5 | 215/216 |  | 99.8 | 482/483 |  | 99.7 | 697/699 |
| IgA or IgG | 99.1 | 214/216 |  | 100 | 483/483 |  | 99.7 | 697/699 |
| IgM or IgG | 98.6 | 213/216 |  | 100 | 483/483 |  | 99.6 | 696/699 |
| IgA or IgM or IgG | 99.5 | 215/216 |  | 100 | 483/483 |  | 99.9 | 698/699 |

**Supplementary Figures**


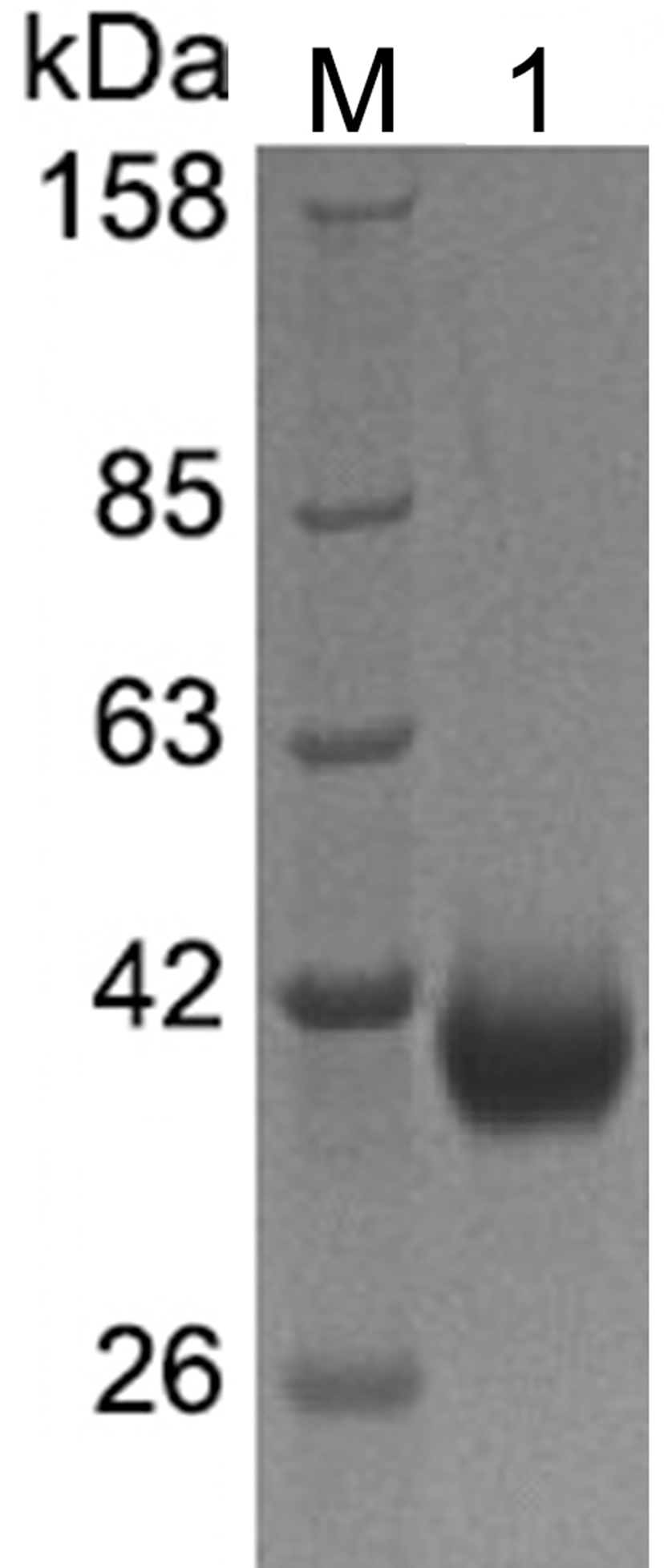


**Supplementary Fig. 1**. Purification of recombinant SARS-CoV-2 receptor-binding domain (RBD) of the spike protein. After SDS-PAGE separation based on molecular weight (kDa) together with protein size markers (M), the proteins were stained with Coomassie Blue. Predicted molecular mass of RBD is 30.3 kDa.


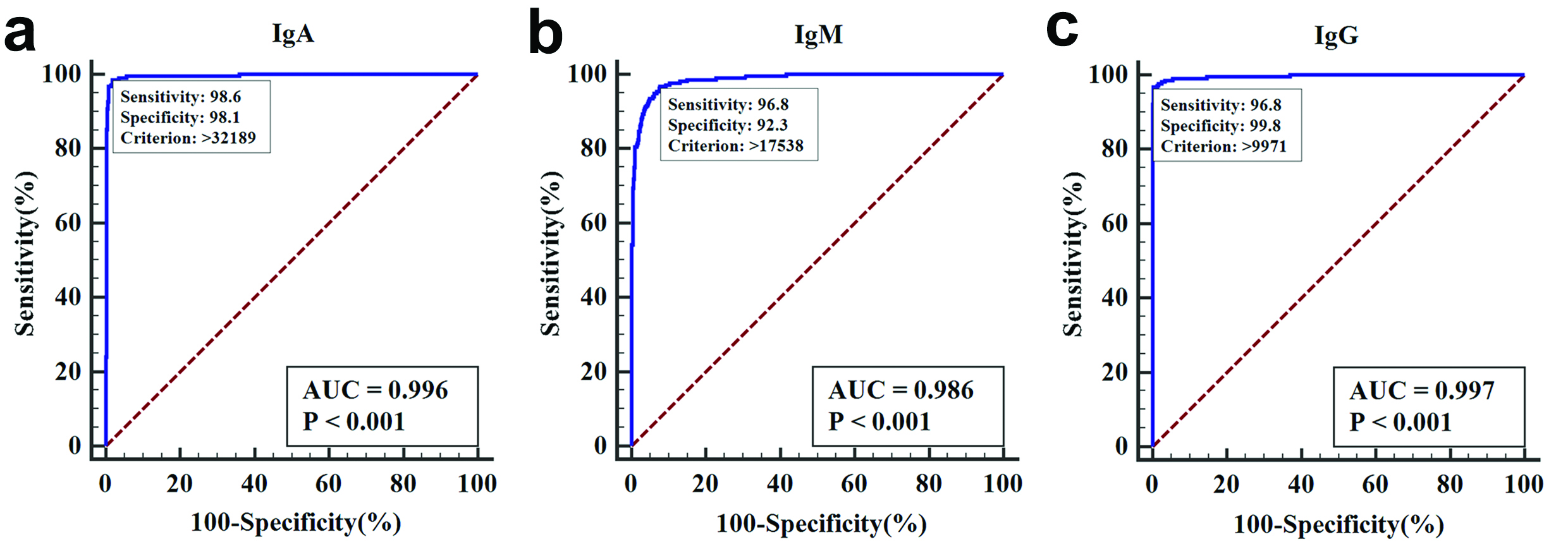


**Supplementary Fig. 2**. The receiver operating characteristic (ROC) curve analysis for SARS-CoV-2 diagnosis. ROC curve analysis for SARS-CoV-2 diagnosis by the testing results of RBD-specific IgA, IgM or IgG (a, b and c, respectively) using 483 sera of SARS-CoV-2 negative individuals as negative controls. AUC, area under the curve of ROC.


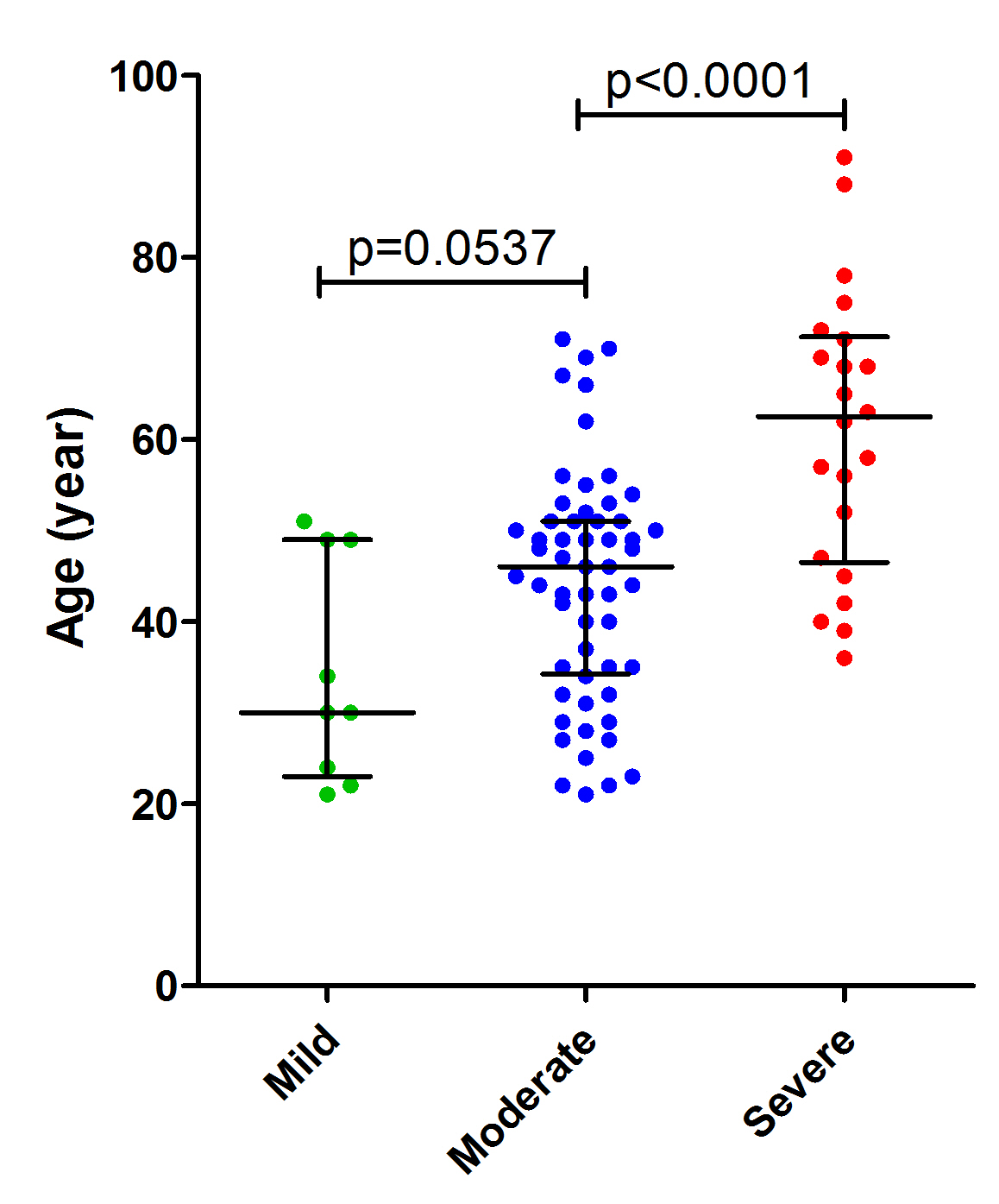


**Supplementary Fig. 3**. Correlation between patient age and disease severity. Mild: 9; moderate: 56; severe: 22 patients.
